# Impact of Surface Roughness on Partition and Selectivity of Ionic Liquids Mixture in Porous Electrode

**DOI:** 10.3390/nano13010051

**Published:** 2022-12-22

**Authors:** Gulou Shen, Haoguang Yang, Yongke Hu, Xiaojie Zhang, Feng Zhou, Huaju Li, Kun Hong

**Affiliations:** National & Local Joint Engineering Research Center for Mineral Salt Deep Utilization, Key Laboratory for Palygorskite Science and Applied Technology of Jiangsu Province, Huaiyin Institute of Technology, Huai’an 223003, China

**Keywords:** ionic liquid, rough electrode, supercapacitors, classical density functional theory

## Abstract

Understanding the influence of surface roughness on the adsorption of ions from an ionic liquids (ILs) mixture is essential for designing supercapacitors. The classical density functional theory (DFT) is applied to investigate the adsorption behavior of ILs mixtures in rough nanopores. The model parameters for each ion are determined by fitting experimental data of pure IL density. The results show that the smaller anions are densely accumulated near the rough surface and are the dominant species at a high positive potential. The exclusion of larger anions is enhanced by roughness at almost all potentials. At negative potential, the surface roughness promotes the adsorption of cations, and the partition coefficient increases with roughness. The partition coefficient of smaller anions is virtually independent of roughness. At positive potential, the surface roughness only promotes the adsorption of smaller anions and raises the partition coefficient. The partition coefficient of smaller anions is far greater than one. The selectivity of smaller anions for rough surfaces is very high and increases with roughness. The surface charge of a more uneven surface is significantly higher (about 30%) at a high potential.

## 1. Introduction

Ionic liquids are liquid salts consisting of organic cations and organic or inorganic anions. They can be in a liquid state below 100 °C. Because of the unique properties of ILs, they have been extensively used in many fields including catalysis, cellulose processing, batteries, supercapacitors, electrodeposition, and lubricant [1,2,3]. The knowledge of solid-IL interface is essential for the applications of ILs. The structure and adsorption of ILs at electrified interfaces significantly influence the performance of ILs in electrochemical processes [4,5].

ILs have been considered suitable electrolytes for supercapacitors that can store electrical energy through the reversible adsorption of ions. The ILs exhibit the electrical double layer (EDL) structure near the charged interface. Numerous studies have focused on the interfacial behavior of ILs near smooth surfaces. However, the solid surface of a porous electrode is usually not perfectly smooth but is somewhat rough. The surface roughness influences the ionic structure and affects the performance of ILs on electrified interfaces. Understanding the partition of ILs in rough nanopores is essential for their use as electrolytes in real applications. Recently some theoretical and simulation work has shown that electrode roughness can improve capacitive performance [6,7,8,9,10]. The surface roughness of porous electrodes has been experimentally shown to enhance the specific capacitance. The molecular dynamics simulations have indicated that the differential capacitance versus surface potential curves at rough surfaces are more complex. 

Mixtures of ILs have gained considerable attention in tuning the properties of ILs. Many IL mixtures have been used in gas absorption, chromatography, liquid-liquid extraction, supercapacitors, and batteries [11,12,13,14,15,16,17,18]. For example, mixing ILs has been demonstrated experimentally to improve the gas absorption performance [13]. IL mixtures have been successfully utilized for dissolution and in situ catalytic degradations of cellulose in a batch reactor [19]. In electrochemistry, some mixing ILs can lead to lower viscosity and conductivity [15]. Recently, for applications of ILs in supercapacitors, the mixture of 1-ethyl-3-methylimidazolium bis(trifluoromethylsulfonyl)imide ([C_2_mim][Tf_2_N]) and 1-ethyl-3-methylimidazolium tetrafluoroborate ([C_2_mim][BF_4_]) have been shown to provide symmetric charge storage and a maximum capacitance, and expand the operational potential window [20,21]. The mixture of tetramethylammonium tetrafluoroborate ([TMA][BF_4_]) and [C_2_mim][BF_4_] can provide simultaneous enhanced power and energy densities [22]. However, the fundamental understanding of mixed ILs in rough nanopores of electrode materials remains limited.

The classical density functional theory (DFT) is a successful and efficient method for studying interfacial properties of ILs at electrified surfaces by taking into account excluded volume effects and electrostatic correlations. The DFT using the fundamental measure theory and mean spherical approximation can reasonably model the EDL structure and capacitance of ILs [21,23,24]. For example, Jiang et al. predicted the capacitance oscillation for [C_2_mim][Tf_2_N] in nanopores [25]. Lian et al. studied the effects of curvature and pore size on the differential and integral capacitances [26]. Liu et al. studied the influence of polar solvents on the performance of IL supercapacitor [27]. Qing et al. studied the charging of asymmetric ILs supercapacitor using a time-dependent DFT [28]. The effects of shape asymmetry and formation of ion pair on ILs EDL have also been studied [29,30,31,32,33,34]. A key point in DFT is to find the accurate approximations of the Helmholtz free energy functional for the inhomogeneous system. A popular class of Helmholtz energy functionals is based on the statistical associating fluid theory (SAFT) equation of state (EoS). The DFT model based on a SAFT EoS can be used to study the bulk and inhomogeneous systems in a consistent way.

In this work, we employ the PC-SAFT-based DFT to study the adsorption and selectivity of mixed ILs in charged rough nanopores, which has rarely been considered in previous studies. We mainly focus on the impact of surface roughness. The Helmholtz energy functional of the DFT model accounts for the hard-sphere, the dispersive, and the electrostatic correlation interactions. A brief overview of the DFT model and a description of the confined system are presented in Section 2. In Section 3, the molecular parameters for each ion are fitted to the experimental density data of pure ILs. The DFT model is applied to explore the impact of roughness on ionic structure, adsorption, and selectivity of the ILs mixtures. Finally, conclusions are given in Section 4.

## 2. Model and Method

In this work, we consider mixtures of [C_2_mim][Tf_2_N] and [C_2_mim][BF_4_] confined in the rough slit-shaped pore of the porous electrode. To model the confined system with PC-SAFT-based DFT, the coarse-grained method is used to represent the ILs. Each IL-ion is modeled as a charged spherical particle of diameter *σ*. The detailed atomistic configuration of ions is not considered. The coarse-grained interaction potential between ions is assumed to be the sum of the soft repulsion of Chen and Kreglewski [35], Lennard-Jones dispersion and electrostatic interaction. The bulk properties of ILs can be calculated using the PC-SAFT-MSA EoS. The two pure IL-ion parameters in the model are the particle-size parameter *σ* and the dispersion-energy parameter *ε*. The Helmholtz free energy of PC-SAFT-MSA EoS is provided in previous publications [32,36].

**Figure 1:**
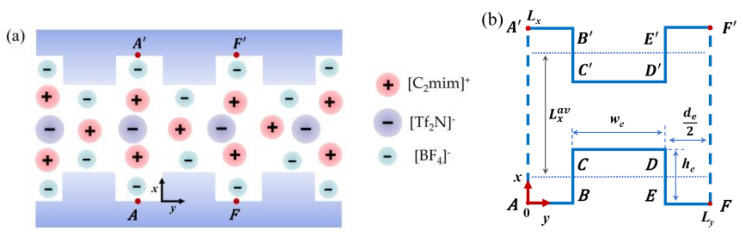
(**a**) A schematic of the structure of IL-ions and a rough nanopore. (**b**) A schematic of the computational domain.

Figure 1a schematically shows the mixture of ILs confined in the rough nanopore. We consider rough walls with arrays of identical square shape roughness elements of width *w_e_* and height *h_e_*. The rough walls are infinite in the y–z plane. The distance between the roughness elements is *d_e_*. 

The nanopore is considered to be an open system in contact with a bulk reservoir. The equilibrium properties of inhomogeneous ILs can be obtained from classical DFT. Therefore, it is convenient to use the grand potential Ω. Ω is a functional of fluid density, and it is written as

(1) $$\Omega =F+\sum_{i}\int dr^{\prime }\rho_{i}\left(r^{\prime }\right)\left(V_{i,ext}\left(r^{\prime }\right)-\mu_{i}\right)$$

In Equation (1), *F* is the Helmholtz energy, $\rho_{i}$ is the density profile of species *i*, and *μ_i_* is the bulk chemical potential. $V_{i,ext}$ is the external potential composed of hard-wall and electrostatic contributions. The ionic profiles are uniform along the z-axis. Thus, we just calculate the two-dimensional ionic profile ρ(x,y). In the y-direction, the external potential is periodic, and the ionic profiles have the period $L_{y}=w_{e}+d_{e}$. We just need to calculate the ionic profiles in the finite range $0\leq y\leq L_{y}$ (see Figure 1b). Figure 1b shows a schematic for the computational domain. The line segments represent the solid wall, such as the line segment CD. The dotted line denotes the average profile of a rough surface. In order to get a better comparison between different surfaces, the pore volume accessible to the ions is a fixed value. The width of the smooth nanopore is equal to $L_{x}^{av}$. The width of a rough nanopore $L_{x}$ is adjusted according to $L_{x}^{av}\left(w_{e}+d_{e}\right)=L_{x}\left(w_{e}+d_{e}\right)-2w_{e}h_{e}$.

The Helmholtz energy functional *F* is a functional of density $\rho_{i}$. *F* can be decomposed into an ideal term *F^id^* and an excess term *F^ex^*

(2) $$F=F^{id}+F^{ex}$$

In Equation (2), *F^ex^* can be decomposed into four terms based on molecular interactions $F^{ex}=F^{hs}+F^{disp}+F^{c}+F^{el}$. *F^c^* denotes the direct Coulomb contribution and is included in the mean electrostatic potential ϕ(r). *F^hs^*, *F^disp^*, and *F^el^* denote hard-sphere repulsion, dispersive attraction, and electrostatic correlation contributions to the functional *F*. These contributions simplify to the corresponding contributions to Helmholtz energy of PC-SAFT-MSA EoS for a homogeneous fluid. Here we briefly introduce the functionals of PC-SAFT-MSA DFT. The modified fundamental measure theory (MFMT) is used to calculate the hard-sphere contribution *F^hs^* [37]

$$\beta F^{hs}=\int dr\Phi^{hs}\left[n_{\alpha }\left(r\right)\right]$$

$\Phi^{hs}$ is the energy density from MFMT, it is a function of six weighted densities $n_{\alpha }\left(r\right)$ proposed by Rosenfeld [38]. The MFMT can accurately describe inhomogeneous hard-sphere fluids.

*F^disp^* is the extension of the dispersive term of PC-SAFT based on the weighted density approximation [34].

$$\beta F^{disp}=\int dr\Phi^{disp}\left[{\bar{\rho }}_{i}\left(r\right)\right]$$

where $\Phi^{disp}$ is similar to the dispersive term of the PC-SAFT, it is a function of averaged densities ${\bar{\rho }}_{i}\left(r\right)$. More details of *F^disp^* are described in previous work [34].

*F^el^* accounts for the electrostatic correlation contribution and is calculated by expanding the functional around the bulk fluid [39].

$$\beta F^{el}=\beta F^{el}\left[\rho_{i,b}\right]-\underset{i}{\sum }\int dr\Delta C_{i}^{1,el}\left(r\right)\left(\rho_{i}\left(r\right)-\rho_{i,b}\right)-\frac{1}{2}\underset{ij}{\sum }\iint drdr^{\prime }\Delta C_{ij}^{2,el}\left(r,r^{\prime }\right)\left(\rho_{i}\left(r\right)-\rho_{i,b}\right)\left(\rho_{j}\left(r\right)-\rho_{i,b}\right)$$

Here, $\rho_{i,b}$ is the bulk density of species *i.* $\Delta C_{i}^{1,el}$ represents the electrostatic contribution to the chemical potential. The expressions of $\Delta C_{ij}^{2,el}$ from the mean spherical approximation are used [40].

In equilibrium, the grand potential is minimal. Thus minimization of Ω with respect to $\rho_{i}$ yields

(3) $$\frac{\delta \Omega }{\delta \rho_{i}\left(r\right)}=0$$

Using Equation (1) and the expressions of F Equation (3) can be explicitly written as

(4) $$\rho_{i}\left(r\right)=\exp\left(\beta \mu_{i}-\frac{\delta \beta \left(F^{hs}+F^{disp}+F^{el}\right)}{\delta \rho_{i}\left(r\right)}-\beta Z_{i}e\phi \left(r\right)-\beta V_{i,ext}^{\prime }\left(r\right)\right)$$

where $\beta =1/k_{B}T$, *k_B_* is the Boltzmann constant, *T* is the temperature, $Z_{i}$ is the valence of component *i*, and *e* is the unit charge. $Z_{i}$ equals +1 for a cation and −1 for an anion. $V_{i,ext}^{\prime }$ is the hard-wall part of $V_{i,ext}$, and the electrostatic part of $V_{i,ext}$ is included in ϕ(r).

For the calculation of the mixture, the Lorentz–Berthelot combining rules are used to obtain the size and energy parameter

$$\{\begin{array}{c} \sigma_{ij}=\frac{\sigma_{i}+\sigma_{j}}{2}\\ \epsilon_{ij}=\sqrt{\varepsilon_{i}\varepsilon_{j}} \end{array}$$

The binary interaction parameter between two components *i* and *j* was not applied.

We have used the fast Fourier transform to calculate the convolution integrals of functional derivatives in Equation (4). The local electrostatic potential ϕ(r) is related to the Poisson equation

(5) $$\nabla^{2}\phi \left(r\right)=\frac{-e}{\varepsilon_{0}\varepsilon_{r}}\sum_{i}Z_{i}\rho_{i}\left(r\right)$$

$\varepsilon_{0}$ is the permittivity of the vacuum. $\varepsilon_{r}$ is the relative dielectric permittivity and is set at 12, this value is close to the experimental data [41]. It is assumed that surface polarization can be ignored.

The Poisson equation with appropriate boundary conditions is discretized using the finite difference method [42]. Equations (4) and (5) are solved self consistently using an iteration method for a specified surface potential $\phi_{s}$. The hybrid algorithm combining Picard iteration and Anderson acceleration is applied to speed up the calculation. The numerical details for DFT calculations can be found in the previous publication [34,43].

From the ionic profiles, we can calculate the total surface charge $Q_{s}$ (C/Å) of a solid surface based on the charge neutrality condition

(6) $$Q_{s}=-\frac{1}{2}\int dxdy\sum_{i}eZ_{i}\rho_{i}\left(x,y\right)$$

The average density ${\bar{\rho }}_{i}$ of species *i* in the nanopore is calculated as

(7) $${\bar{\rho }}_{i}=\frac{1}{L_{x}L_{y}-2w_{p}h_{p}}\int dxdy\rho_{i}\left(x,y\right)$$

The partition coefficient is calculated as

(8) $$\Gamma_{i}=\frac{{\bar{\rho }}_{i}}{\rho_{i,b}}$$

The selectivity of the smaller anion [BF_4_]^−^ over the larger anion [Tf_2_N]^−^ is defined as $S=\Gamma_{\mathrm{BF}4}/\Gamma_{\mathrm{Tf}2N}$.

## 3. Results and Discussion

### 3.1. Bulk Density of Pure Ionic Liquids and Their Mixtures

**Figure 2:**
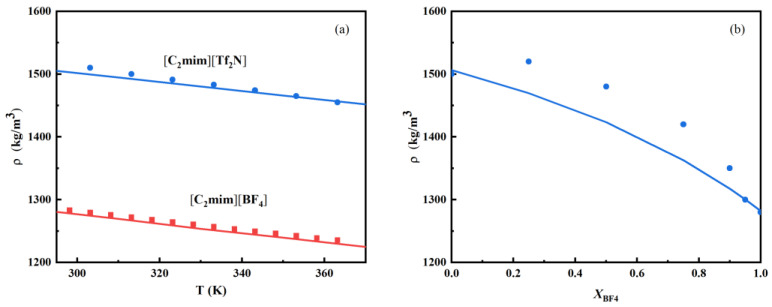
(**a**) Bulk density of pure ILs [C_2_mim][Tf_2_N] and [C_2_mim][BF_4_] at T = 293.1K and atmospheric pressure. Symbols: experimental data [48,49]; lines: model results. (**b**) Density of mixture [C_2_mim][Tf_2_N]/[C_2_mim][BF_4_] as a function of mole fraction of [C_2_mim][BF_4_] (*X_BF4_*), T = 293.1K. Symbols: experimental data [47]; line: model prediction.

In order to determine the model parameters for each ion, we first apply the PC-SAFT-MSA EoS to model the bulk density of pure ILs. The ionic parameters *σ* and *ε* are fitted to the experimental data of density of [C_2_mim][Tf_2_N] and [C_2_mim][BF_4_] [44,45,46]. The parameters are $\sigma_{C2\mathrm{mim}}=5.25\;Å$ and ${\left(\varepsilon /k_{B}\right)}_{C2\mathrm{mim}}=697.77\;K$, $\sigma_{\mathrm{Tf}2N}=6.44\;Å$ and ${\left(\varepsilon /k_{B}\right)}_{\mathrm{Tf}2N}=855.12\;K$, $\sigma_{\mathrm{BF}4}=4.54\;Å$ and ${\left(\varepsilon /k_{B}\right)}_{\mathrm{BF}4}=597.15\;K$. The absolute average relative deviation (AARD) is considered. The deviation is defined by $\mathrm{AARD}=\frac{100}{N}\overset{N}{\underset{i=1}{\sum }}\left|\rho^{cal}-\rho^{exp}\right|/\rho^{exp}$, $\rho^{cal}$ and $\rho^{exp}$ are the calculated density and experimental density, respectively. The AARD of 0.5% is obtained for pure ILs density. Figure 2a shows an example of model results at atmospheric pressure. The symbols denote experimentally measured density, and the lines indicate the model result. The model result is in good agreement with the experimental data.

Then, the ionic parameters are used to predict the density of mixtures of [C_2_mim][Tf_2_N]/[C_2_mim][BF_4_] without any binary parameter. Figure 2b shows an example of model results at a temperature of 293.1K and atmospheric pressure. The symbols are experimental data [47], and the line represents model prediction. The model prediction agrees with experimental data, and the AARD is about 2%. We have also calculated the relative deviation between the predicted density and experimental data for each composition, the result is shown in Figure S1 in the supplementary material. The maximum relative deviation is about 4%.

### 3.2. Ionic Liquids Mixtures in Rough Nanopores

In this section, the DFT model is used to study mixtures of ILs confined in the rough pore. The temperature and the pressure are fixed at 323 K and 1 bar. Two ILs mixtures were considered in this work with molar fraction of [C_2_mim][BF_4_] in the bulk phase *X_BF4_* equals 0.2 and 0.5, respectively. The calculated number density of the mixture in the bulk phase is 4.97 nm^−3^ for *X_BF4_* = 0.2 and 5.73 nm^−3^ for *X_BF4_* = 0.5. For rough pores, the roughness width *w_e_* is set at 1.4 nm, and the distance between roughness element *d_e_* = *w_e_*. The average size of the pore $L_{x}^{av}$ is set at 3 nm. The grid resolution of the two-dimensional DFT computation is 0.01 nm. 

We have studied the adsorption of mixed ILs in nanopores with roughness height *h_e_* ranging from 0 to 0.56 nm. The surface potential $\phi_{s}$ varies from −1 V to 1 V. The Wenzel roughness factor is an important parameter to characterize a rough surface. The Wenzel roughness factor is the ratio of the true area to the apparent area of the solid surface [50]. Thus the roughness factor for the considered surface can be calculated as $r_{e}=1+2h_{e}/\left(w_{e}+d_{e}\right)$. For a smooth surface, *r_e_* equals one. For a rough surface, *r_e_* is greater than one and increases with *h_e_*.

**Figure 3:**
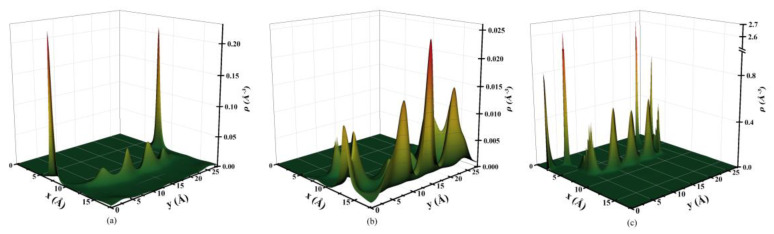
Two-dimensional density profiles of [C_2_mim]^+^ (**a**), [Tf_2_N]^−^ (**b**), and [BF_4_]^−^ (**c**) in a nanopore at surface potential $\phi_{s}=1\;V$, *X_BF4_* = 0.2, and $r_{e}=1.4$ that corresponds to *h_e_* = 0.56 nm.

**Figure 4:**
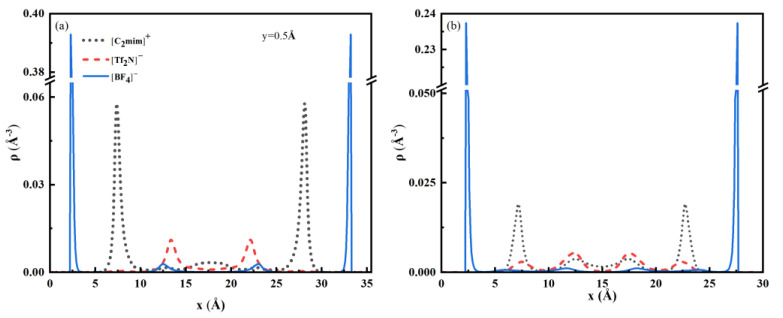
(**a**) One-dimensional density curves of ions taken at the specified y position. Other conditions are the same as in Figure 3. (**b**) Density profiles for ions in a smooth pore at surface potential $\phi_{s}=1\;V$, and *X_BF4_* = 0.2.

We first investigate the two-dimensional ion structure in rough nanopores. The rough walls induce oscillations in the density profiles in both normal (perpendicular to the substrate wall) and lateral (parallel to the substrate wall) directions. For example, Figure 3 and Figure 4 show two-dimensional density profiles for ions in a rough pore with the roughness factor $r_{e}=1.4$, several normal density curves taken at the specified location, as well as the density profiles for ions in the smooth pore. More examples of normal and lateral density curves are shown in Figure S2. The molar fraction of [BF_4_]^−^ in the bulk ILs mixture is *X_BF4_* = 0.2, and the surface potential $\phi_{s}$ is 1 V. The smaller anions are densely packed near the rough surface with a significant lateral structure, and the density profile shows sharp peaks. The larger anions and cations are excluded from the surface. 

In the x direction, the ions have a multilayer EDL structure. The smaller anions can get closer to the surface and occupy a smaller space, and they are more widely distributed in the pore. The contact density of smaller anions around the rough wall is higher than that around the smooth wall. The larger anions are mainly located near the middle of the pore, and almost none of them exist at the surface. Thus, at high surface potential, the smaller anions are more strongly adsorbed to the surface. In the y direction, at the bottom (line segment AB as shown in Figure 1b) and top (line segment CD) of the rough surface, there is considerable structure. As one can see in Figure 3c, there are three strong layers of smaller anions [BF_4_]^−^ at the bottom of the surface, the layer thickness is of the same order as the anion’s diameter. There are almost no cations and larger anions in this region. To gain a further understanding of the structure, we investigate the local excess adsorption, which is calculated as

$$N_{i}\left(x\right)=\int dy\left(\rho_{i}\left(x,y\right)-\rho_{i,b}\right)$$

**Figure 5:**
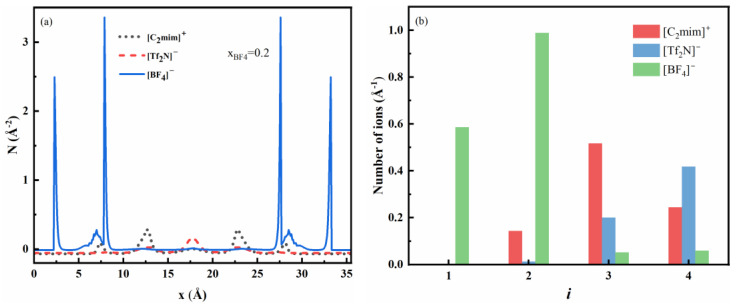
(**a**) Local excess adsorption of ions in nanopore with rough surfaces. (**b**) Number of ions adsorbed in the four layers near the surface (*i* = 1 is the first layer, *i* = 2 is the second layer, *i* = 3 is the third layer, *i* = 4 is the fourth layer). Other conditions are the same as in Figure 3.

We have calculated the local excess adsorptions of three ions for two bulk concentrations, Figure 5a shows the result for bulk concentration *X_BF4_* = 0.2, and the result for *X_BF4_* = 0.5 is shown in Figure S3, all other parameters are the same as in Figure 3. There are four layers of excess ions near a surface. If we calculate the integral of ionic density in the layer we get the number of adsorbed ions. Figure 5b shows the number of ions adsorbed in each layer. The excess adsorption of smaller anions is higher and exhibits two peaks near the solid wall, and the small anions are the dominant species in the near-wall region. The first peak indicates the adsorption of small anions at the bottom of rough surface (line segments AB and EF), and the second peak indicates the adsorption around the top of rough surface (line segment CD). The smaller anions accumulate in the first and second layers and have high density, even at a low bulk concentration. The excess adsorption of smaller anions away from the wall is close to zero, and the local average density will be almost the same as the bulk value. When the bulk composition of smaller anions increases to 0.5, the value of excess adsorption peak for smaller ions around the wall shows little change. The number of larger anions in the first and second layers is close to zero and the total number of larger anions is much lower than smaller anions. Therefore, the larger anions tend to be excluded from the pore, and they are only enriched in the central region of the pore.

**Figure 6:**
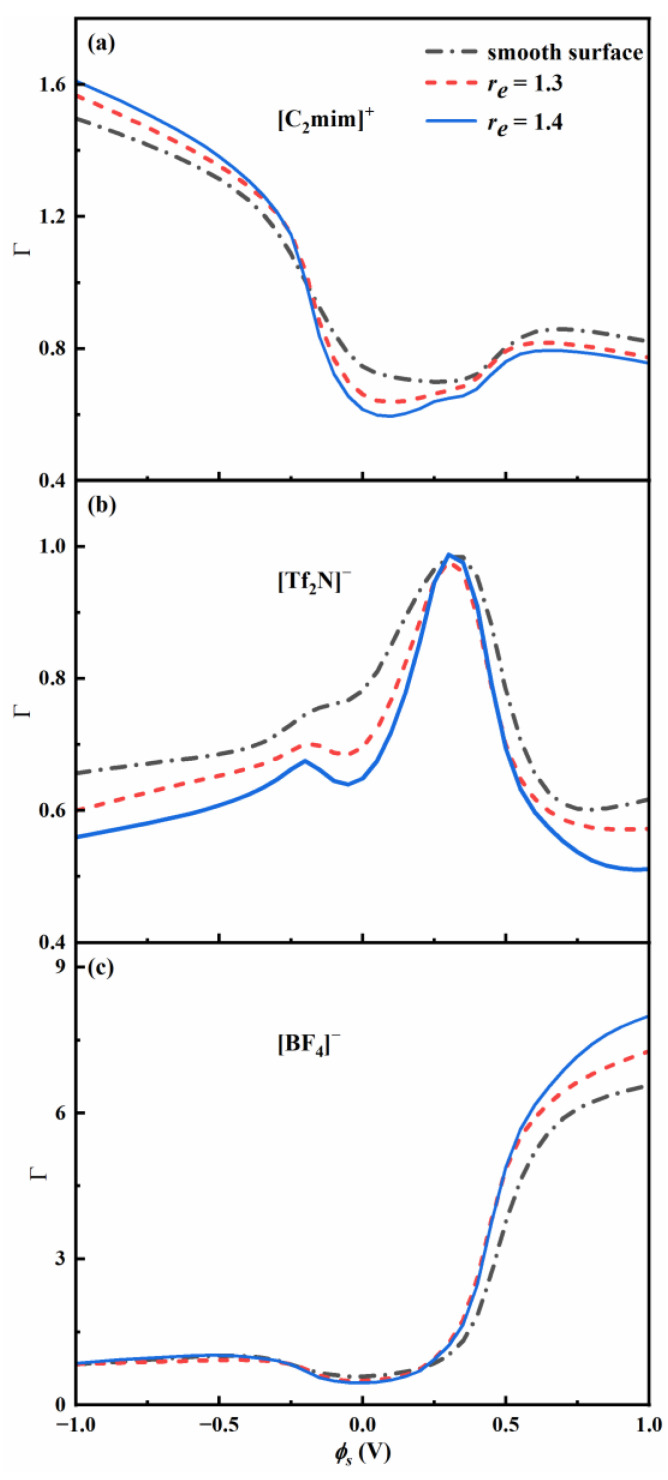
Partition coefficient of ions as a function of $\phi_{s}$, the bulk molar fraction *X_BF4_* is 0.2.

Then, we investigate the effect of surface roughness on the adsorption and selectivity of ions. Figure 6 shows the calculated partition coefficient of ions as a function of $\phi_{s}$, and the corresponding average density of each ion adsorbed inside the pore is shown in Figure S4, the bulk composition *X_BF4_* is 0.2, and roughness factor $r_{e}$ = 1, 1.3 and 1.4. The partition coefficient is directly proportional to the average density as shown in Equation (8). At low absolute potential, the increasing surface roughness results in a decrease in the average density of cations and larger anions, so cations and larger anions tend to be excluded from the rough pores. $\bar{\rho }$ of larger anions [Tf_2_N]^−^ is significantly greater than that of smaller anions [BF_4_]^−^ because there are more anions [Tf_2_N]^−^ in the connected bulk phase. For the bulk composition *X_BF4_* = 0.5, $\bar{\rho }$ of larger anions is only slightly greater than that of smaller anions. 

At negative potential, the cations become enriched in the nanopore, and their average density monotonically increases with absolute potential. When the surfaces are sufficiently negatively charged, $\bar{\rho }$ of all anions decreases with increasing voltage. As the surface roughness increases, $\bar{\rho }$ of cations increases, and $\bar{\rho }$ of larger anions [Tf_2_N]^−^ decreases. Nevertheless, the average density of smaller anions [BF_4_]^−^ is almost independent of surface roughness. Overall, the adsorption of cations and the exclusion of larger anions are significantly enhanced by roughness. 

At positive potential, the anions become enriched in the nanopore. As the surface roughness becomes higher, $\bar{\rho }$ of smaller anions monotonically increases, whereas the $\bar{\rho }$ of larger anions first increases, passes through a maximum, and then decreases. As the surface roughness increases, $\bar{\rho }$ of cations and larger anions [Tf_2_N]^−^ decreases, while $\bar{\rho }$ of smaller anions [BF_4_]^−^ significantly increases. Thus, the surface roughness induces exclusion toward larger anions in almost the entire range of potentials. The adsorption of smaller anions is promoted by roughness at positive potentials and is more energetically favorable than that of larger anions. 

At low potential, $\bar{\rho }$ of all ions is lower than the bulk density, so all ions tend to be excluded from the nanopore. $\bar{\rho }$ of larger anions [Tf_2_N]^−^ is lower than the bulk density in the entire range of potentials. Thus, the partition coefficient of larger anions is less than one and decreases with increasing surface roughness.

At high positive potential, the high value of $\bar{\rho }$ of smaller anions indicates that smaller anions neutralize the surface charge favorably. $\bar{\rho }$ of smaller anions [BF_4_]^−^ is much higher than the bulk density. Thus, the partition coefficient of smaller anions is far greater than one and increases with increasing surface roughness. Table 1 and Table 2 show the partition coefficient and selectivity of smaller anions [BF_4_]^−^ for two bulk compositions, the potential $\phi_{s}$ is 1 V. For the bulk composition *X_BF4_* = 0.5, the partition coefficient of smaller anions at high positive potential is significantly lower (around 3). 

At high negative potential, the partition coefficient of cations is greater than one, and is slightly lower for *X_BF4_* = 0.5. The partition coefficient of smaller anions is virtually independent of roughness and slightly decreases with increasing absolute potential.

**Figure 7:**
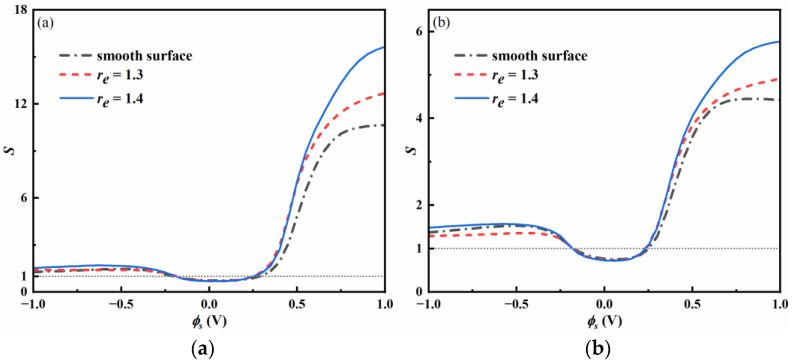
Selectivity of [BF_4_]^−^ over [Tf_2_N]^−^ as a function of $\phi_{s}$. (**a**) *X_BF4_* = 0.2 and (**b**) *X_BF4_* = 0.5.

Figure 7 shows the selectivity of [BF_4_]^−^ over [Tf_2_N]^−^ as a function of surface potential for two bulk compositions. At low absolute potential, the selectivity *S* < 1, larger anions are preferred inside the pore. The surface roughness does not influence the selectivity.

When the surface is strongly negative-charged, the selectivity *S* > 1, and the smaller anions [BF_4_]^−^ are selected. A similar trend has been reported by Neal et al. for ILs mixture in smooth nanopores [51]. 

At moderate and high positive potential, adsorption of the smaller anions [BF_4_]^−^ is energetically more favorable, so the selectivity *S* > 1. The rough surfaces can enhance the adsorption of smaller anions, a significant selectivity for rough surfaces is observed, and the selectivity increases with increasing surface potential. The selectivity for rough surfaces with *r_e_* = 1.4 is almost 1.6 times the value for smooth surfaces. The selectivity is lower for a higher bulk composition of [BF_4_]^−^.

Overall, at high potential, the electrosorption of small anions inside the pore is more energetically favorable. An increase in surface roughness leads to an increase in selectivity.

**Figure 8:**
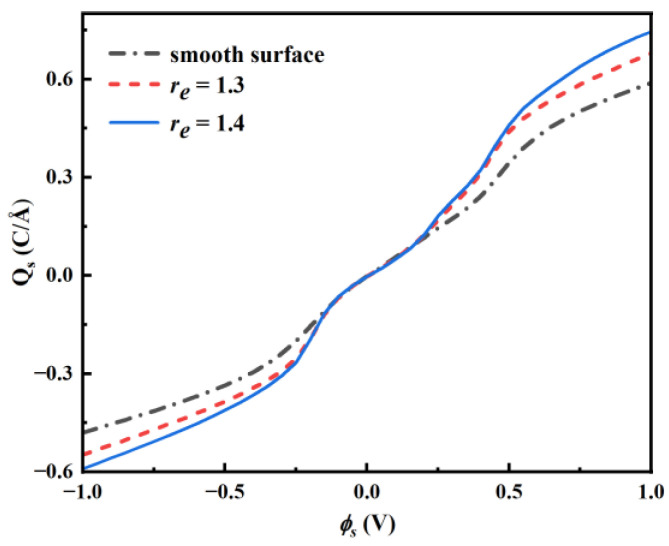
Surface charge as a function of $\phi_{s}$, *X_BF4_* = 0.2.

In the end, the surface charge $Q_{s}$ is investigated. For example, Figure 8 shows the surface charge versus $\phi_{s}$ for *X_BF4_* = 0.2. $Q_{s}$ monotonically increases with $\phi_{s}$. When the absolute value of $\phi_{s}$ is high, the absolute value of $Q_{s}$ of a rough pore is considerably higher than that of a smooth pore. The absolute surface charge is higher for higher roughness. Because the volumes of different walls are alike, the rough pore tends to give a higher volume-specific integral capacitance. 

Overall, the increase in surface roughness also induces a rise in the absolute surface charge. As the roughness increases, the reason for increasing absolute surface charge at negative potential is that the adsorption of cations and exclusion of larger anions are enhanced. The reason for increasing surface charge at positive potential is that the adsorption of smaller anions and exclusion of cations are enhanced.

## 4. Conclusions

In this work, we have investigated the adsorption and selectivity of mixed ILs in rough nanopores through a PC-SAFT-MSA-based DFT. The molecular parameters of ILs are fitted to the experimental data of pure liquid density. The molecular parameters are used to predict the bulk density of mixed ILs. The predicted density agrees with experimental data. For the confined system, the results show that at a high positive potential, the smaller anions are densely accumulated near the rough surface and are the dominant species. The larger anions tend to be excluded from the pore. The surface roughness enhances the exclusion of larger anions in the entire range of potentials, and the partition coefficient decreases with increasing roughness. At negative potential, the adsorption of cations is promoted by surface roughness, and the partition coefficient increases with increasing roughness. However, the surface roughness does not affect the adsorption of smaller anions; thus, the partition coefficient of smaller anions is independent of roughness. At positive potential, the surface roughness significantly enhances the adsorption of smaller anions. The partition coefficient of smaller anions is far greater than one and increases with surface roughness. The selectivity of smaller anions for a rough pore is higher than for a smooth pore and increases with roughness. At high surface potential, the absolute value of the surface charge of a rough pore is significantly larger than that of a smooth pore. Therefore, the rough electrode can hold more charge.

This work gives insight into the selective adsorption of mixed ILs in rough nanopores and helps to understand the charge storage on real mixed ILs/electrode interface. The results indicate that both composition and atomic scale roughness of surface are important factors that have to be considered for the optimization of ILs capacitors. In the present study, some other factors that may impact the partition and selectivity of mixed ILs are neglected, for example, the atomic configuration of ions, surface polarization, and the association between the cation and the anion. The IL is fully dissociated in the model. Therefore, the model needs to be further improved for some protic ILs that contain neutral species.

## Figures and Tables

**Table 1:** Partition coefficient and selectivity of [BF4]^−^, $\phi_{s}=1\;V$ and *X_BF4_* = 0.2.

		*r_e_*	
	1	1.3	1.4
*Γ*_BF4_	6.6	7.3	8
*S*	10.7	12.7	15.7

**Table 2:** Partition coefficient and selectivity of [BF4]^−^, $\phi_{s}=1\;V$ and *X_BF4_* = 0.5.

		*r_e_*	
	1	1.3	1.4
*Γ*_BF4_	2.7	2.9	3.1
*S*	4.4	4.9	5.8

## Data Availability

The data that support the findings of this study are available within the article.

## Supplementary Materials

The following supporting information can be downloaded at: https://www.mdpi.com/article/10.3390/nano13010051/s1, Figure S1: Relative deviation for density of mixture [C_2_mim][Tf_2_N]/[C_2_mim][BF_4_]. Relative deviation is calculated as $\left(\rho^{cal}-\rho^{exp}\right)/\rho^{exp}$; Figure S2: One-dimensional density curves of ions taken at the specified x and y positions. Other conditions are the same as in Figure 3; Figure S3: Local excess adsorption of ions in nanopore with rough surfaces, *X_BF4_* = 0.5, other conditions are the same as in Figure 3; Figure S4: Average density of ions as a function of $\phi_{s}$, the bulk molar fraction *X_BF4_* is 0.2.
