# Supplementary material for: Impact of Surface Roughness on Partition and Selectivity of Ionic Liquids Mixture in Porous Electrode
Source: Nanomaterials (Basel). 2022 Dec 22;13(1):51. doi: 10.3390/nano13010051 (PMC9823643; doi:10.3390/nano13010051)
Supplement: Supplementary file 1 [file nanomaterials-13-00051-s001.zip › nanomaterials-2103665-supplementary.pdf]

nanomaterials

Supplementary Materials

Impact of Surface Roughness on Partition and Selectivity of Ionic Liquids Mixture in Porous Electrode

Gulou Shen *, Haoguang Yang, Yongke Hu, Xiaojie Zhang, Feng Zhou, Huaju Li and Kun Hong

National & Local Joint Engineering Research Center for Mineral Salt Deep Utilization, Key Laboratory for Palygorskite Science and Applied Technology of Jiangsu Province, Huaiyin Institute of Technology, Huai'an 223003, China
* Correspondence: lsheng@hyit.edu.cn

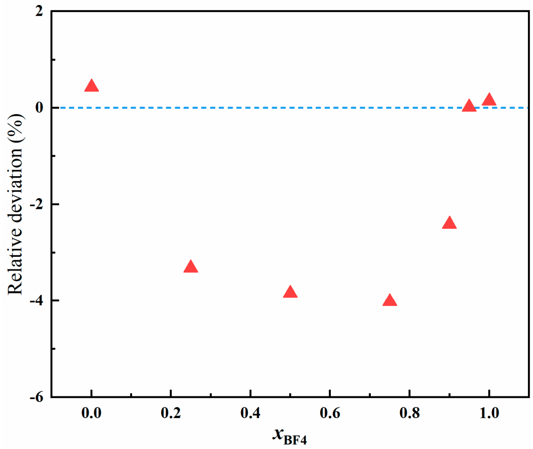

Figure S1. Relative deviation for density of mixture $[C_2mim][Tf_2N]$/$[C_2mim][BF_4]$. Relative deviation is calculated as $(\rho^{cal} - \rho^{exp})/\rho^{exp}$.

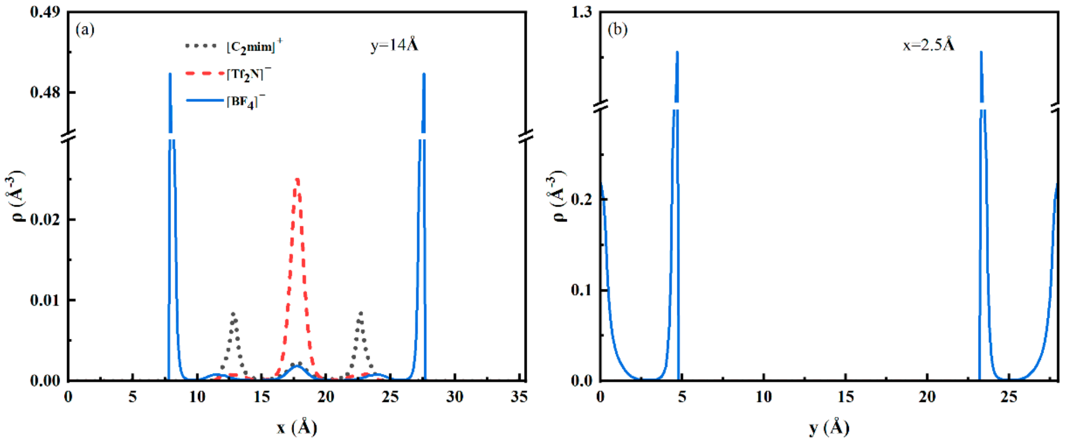

Figure S2. One-dimensional density curves of ions taken at the specified x and y positions. Other conditions are the same as in Figure 3.

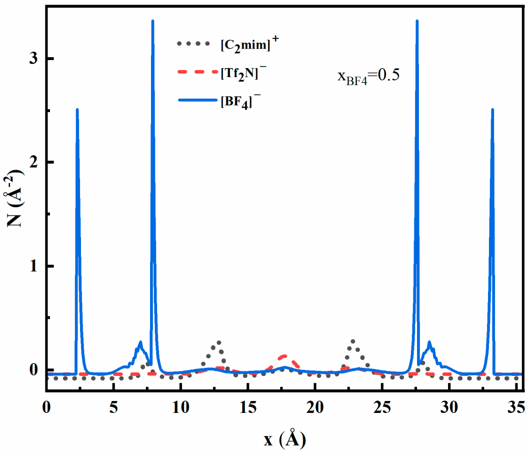

Figure S3. Local excess adsorption of ions in nanopore with rough surfaces, X_{BF4} = 0.5, other conditions are the same as in Figure 3.

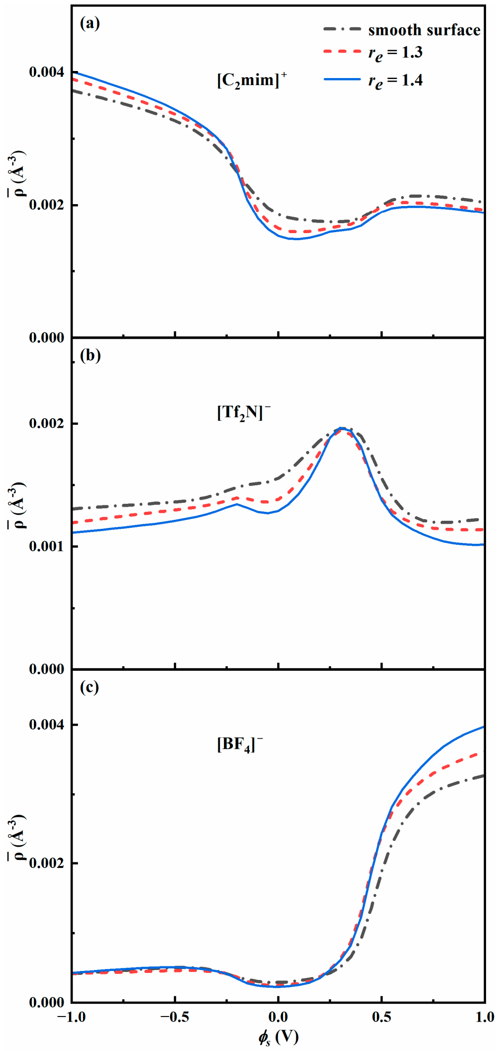

Figure S4. Average density of ions as a function of ϕ_s, the bulk molar fraction X_{BF4} is 0.2.
